# Characterization and Effect of Thermal Annealing on InAs Quantum Dots Grown by Droplet Epitaxy on GaAs(111)A Substrates

**DOI:** 10.1186/s11671-015-0930-3

**Published:** 2015-06-02

**Authors:** Sergio Bietti, Luca Esposito, Alexey Fedorov, Andrea Ballabio, Andrea Martinelli, Stefano Sanguinetti

**Affiliations:** L-NESS and Dipartimento di Scienza dei Materiali, Università di Milano-Bicocca, via Cozzi 55, Milano, I–20125 Italy; CNR–IFN and L–NESS, via Anzani 42, Como, I–22100 Italy

**Keywords:** Droplet epitaxy, InAs quantum dots, GaAs(111)A

## Abstract

We report the study on formation and thermal annealing of InAs quantum dots grown by droplet epitaxy on GaAs (111)A surface. By following the changes in RHEED pattern, we found that InAs quantum dots arsenized at low temperature are lattice matched with GaAs substrate, becoming almost fully relaxed when substrate temperature is increased. Morphological characterizations performed by atomic force microscopy show that annealing process is able to change density and aspect ratio of InAs quantum dots and also to narrow size distribution.

## Background

The self–assembly of quantum dots (QDs) attracts a great interest for the possibility to fabricate advanced photoelectronic devices such as single photon emitters and entangled photon sources [1, 2]. In particular, droplet epitaxy (DE) technique [3–5] has recently demonstrated the possibility to grow high-quality quantum nanostructures in lattice-matched and mismatched systems with a high degree of control over density, size, and shape of the nanostructures [6–11] suitable for the fabrication of single photon emitters at liquid nitrogen temperature and entangled photon sources [12–14]. The flexibility of DE is due to the fact that the growth of III-V QDs is performed in two distinct steps. In the first one, the group-III element is deposited on the substrate to form liquid droplets; in the second step, a flux of group-V element is irradiated in order to crystallize the droplets in quantum nanostructures.

In order to shift the QD-DE emission towards telecommunication wavelength range (1.3–1.5 *μ*m) and to reduce the fine structure splitting (FSS), many efforts have been devoted to the fabrication of InAs QDs on GaAs(111) surfaces [14–17]. Compared to (100), (111) surface is of extreme interest due to the fact that the C _3*v*_ symmetry of (111) surface allows to realize highly symmetric QDs with a vanishing FSS [15]. Unfortunately, fabrication of InAs QDs on GaAs(111) by Stranski–Krastanow growth mode is impossible because strain relaxation takes place by the introduction of dislocations instead of three-dimensional island formation [18–20]. Recently, GaAs QDs grown by DE on GaAs(111)A surface were fabricated [21, 22] and control of nuclear spin [23], charge tuning [24], magneto-optical properties [25], interplay between exchange and Zeeman effect [26], and emission of entangled photon pairs [27] were studied. Successful deposition of InAs QDs by DE on GaAs(100) surface have been reported in [28–30], while recently studies about the formation and morphology of InAs QDs were reported in [31, 32].

Despite this interest, only few works showed the possibility to grow In(Ga)As QDs on (111) surfaces. Single photon emission is reported in [16], and a reduced FSS for entangled photon emission near telecommunication wavelength ranges is reported in [14]. Anyway, a study on formation and morphology of InAs QDs grown on GaAs(111)A is not available in scientific literature.

In this work, we report the analysis of the different steps for the growth of InAs QDs by DE technique on GaAs(111)A surface by reflection high-energy electron diffraction (RHEED) to better understand the growth mechanism of InAs QDs, and we analyze the effect of annealing on density and size distribution of QDs by changing the initial size of the QDs and the annealing temperature. The annealing process is a step necessary to remove As excess on the surface exposed to high As flux at low temperature and in particular for QDs formed at low temperature in order to improve the crystalline quality [33–35]. In our experiments, we found evidence that the annealing process changes the density and the aspect ratio (the ratio between the height and the diameter of a QD) of InAs islands, narrowing the size distribution. We also observed from RHEED pattern that it is possible to fully convert a solid In nano–crystal into an InAs nano–crystal pseudomorphic with the GaAs substrate, becoming almost fully relaxed when substrate temperature is increased up to 300 °C.

## Methods

Two series of samples were grown on GaAs (111)A substrates inside a Gen II MBE chamber with an As valved cracker cell. All the different steps for the growth were monitored with a RHEED system. The electron beam was generated inside a VG LEG 110 RHEED gun and acquired by a CCD camera in front of a fluorescent screen. After oxide removal, a GaAs buffer layer was deposited to obtain a flat surface. The substrate temperature was then decreased to 350 °C, and As pressure in the chamber reduced below 10^−9^ torr. Indium droplets were deposited at the same temperature with a beam flux of 3.5×10^13^ atoms s ^−1^ cm ^−2^. Substrate temperature was then decreased to 100 °C and then an As _4_ flux of 7.2×10^15^ atoms s ^−1^ cm ^−2^ supplied for 3 min. Finally, the samples were submitted to a thermal anneal treatment in As flux of 7.2×10^15^ atoms s ^−1^ cm ^−2^ for 3 min. Two series of samples were grown by depositing different amounts of In and by changing substrate temperature during annealing procedure. Table 1 reports the different conditions for the fabrication of the two series of samples. After the growth, the samples were analyzed by an atomic force microscope (AFM) in tapping mode, using a ultra-sharp tip, capable of a lateral resolution of about 2 nm.

**Table 1:** Growth parameters and morphological data for the two sets of samples: In amount deposited (here is reported the equivalent amount on GaAs(100) surface), substrate temperature during the annealing procedure, density of InAs QDs, percentage of deposited In incorporated in InAs QDs, mean value of radius, mean value of aspect ratio

Sample	In amount	T annealing	QD density	% of In deposited	Mean R	Mean AR
	(ML)	(°C)	(×10^8^ cm ^−2^)	incorporated in QDs	(nm)	
L1	1.5	300	161.2	100.0	25.2 ± 4.9	0.123 ± 0.015
L2	0.6	300	5.1	0.7	18.4 ± 4.7	0.055 ± 0.008
L3	0.4	300	0	0.0	-	-
H1	1.7	450	18.6	4.1	24.0 ± 2.6	0.084 ± 0.028
H2	1.5	450	10.4	2.9	22.9 ± 3.1	0.062 ± 0.015
H3	1.0	450	0	0.0	-	-

## Results and Discussion

### QD Formation

**Fig. 1:**
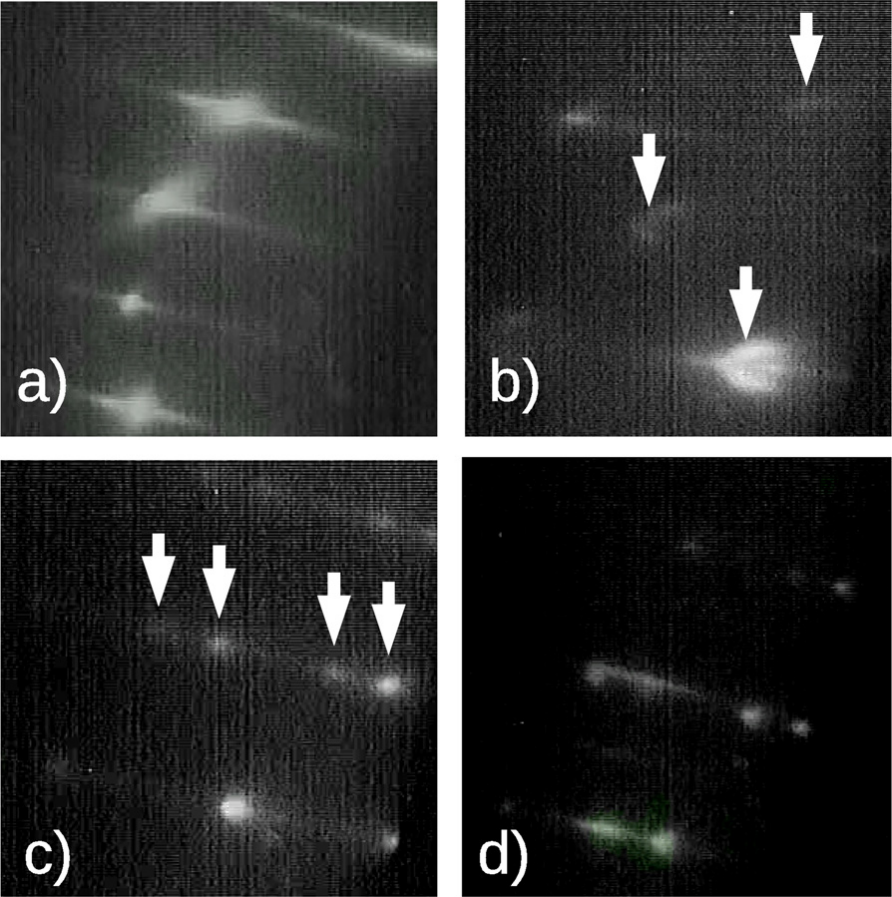
RHEED pattern during the growth of InAs QDs on sample L1. **a** (2×2) reconstruction of GaAs(111)A along $[21\bar {1}]$ direction before In deposition. **b** GaAs surface along $[21\bar {1}]$ direction at 100 °C showing presence of crystalline In spots (evidenced by arrows). **c** InAs QD spots along [011] direction at low temperature during arsenization showing a lattice matched InAs dots on GaAs(111)A surface. Arrows evidence the presence of twins. **d** InAs QD spots along [011] direction after annealing at 300 °C showing relaxation of InAs QDs

**Fig. 2:**
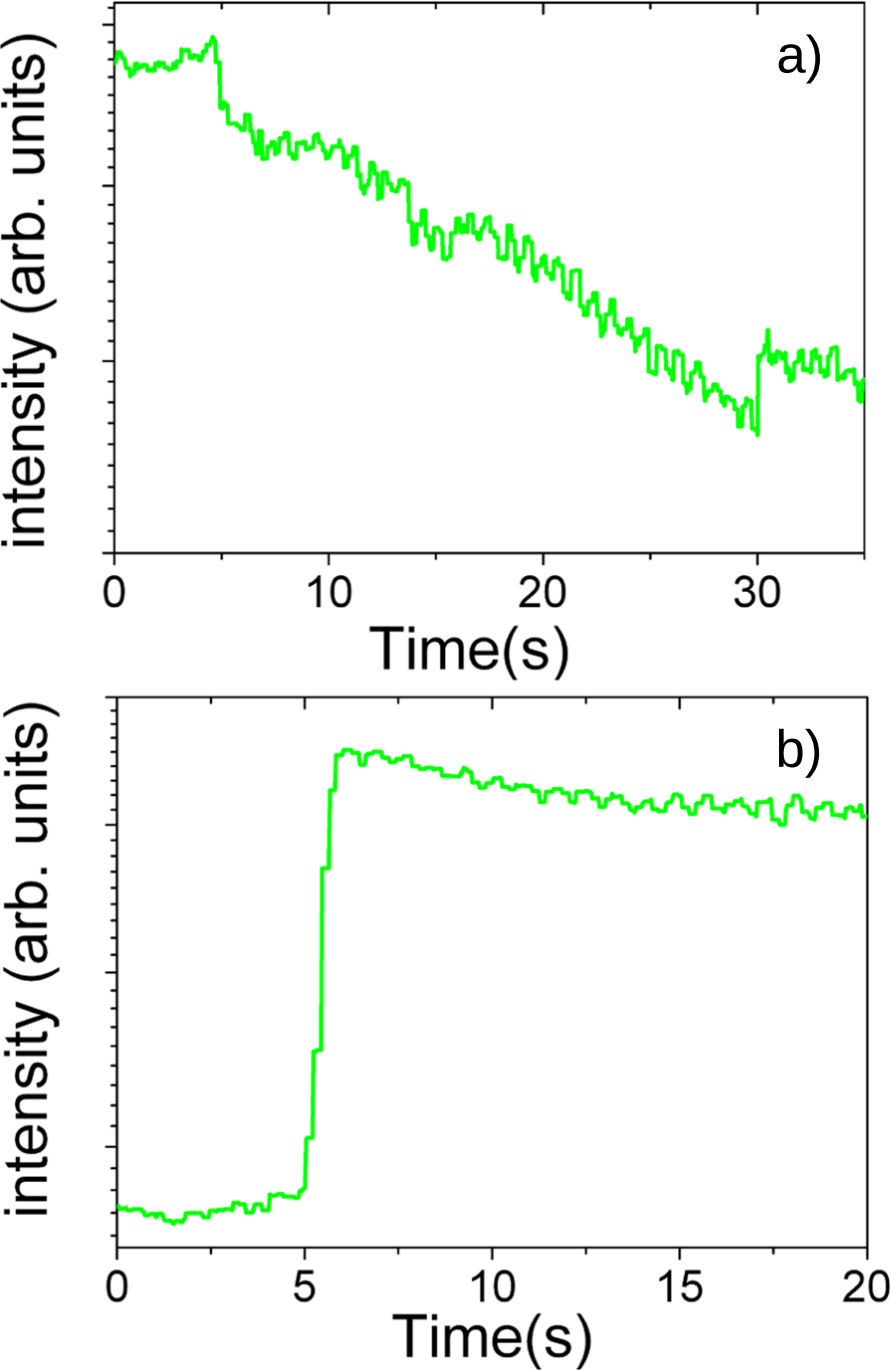
RHEED intensity change during In deposition on GaAs(111)A surface observed on a GaAs streak (**a**) and during In island arsenization observed on transmission spot (**b**)

The RHEED changes observed during the growth of sample L1 are reported in Fig. 1. Figure 1a shows (2×2) reconstruction observed on GaAs(111)A surface along $[21\bar {1}]$ direction before In deposition. The formation of a (2×2) reconstruction is expected, as reported in [36, 37]. During In deposition, intensity of the streaks slightly faded, due to the presence of liquid In droplets, which act as electron scatterers on GaAs surface. The variation in intensity of specular beam during In deposition is reported in Fig. 2a. After In shutter is opened, intensity decreases for the whole duration of In deposition. No additional features appeared in RHEED pattern. This behavior is in agreement with the data reported in DE experiments performed depositing Ga droplets on GaAs (001) surface [3]. Unlike the case of GaAs(001), where surface is typically terminated with an excess of As, on GaAs(111)A droplets nucleates immediately on Ga-terminated surface [36]. In Fig. 1b, RHEED pattern observed along $[21\bar {1}]$ direction after reducing substrate temperature to 100 °C and before As irradiation is reported. It is important to notice that during this step, substrate temperature is below melting point of In. In this pattern, the ratio between spacing of V-shaped spots (indicated by a white arrow in Fig. 1b) and the one of streaks related to GaAs (111)A surface is in good agreement with the inverse of the ratio between the distance of GaAs planes and the cell parameter of In crystalline structure [38]. The presence of these spots can be attributed to body centred tetragonal structure of solid In. The chevron shape of the spots is related to the formation of facets [38] on In nano–crystals. The evolution of RHEED pattern thus demonstrates that Indium is liquid during deposition at 350 °C and turns into a epitaxial crystalline solid when substrate temperature is reduced, as also reported for In deposition on GaAs(001) [31]. Figure 1c reports the RHEED pattern observed along [011] direction immediately after As flux irradiation. The RHEED pattern changes again in less than one second (intensity variation of a transmission spot is reported in Fig. 2b). This is the fingerprint of formation of InAs QDs [3–5]. Despite the presence of In in crystalline phase, the time required for the arsenization process is in agreement with the one observed for liquid Ga droplets (see [4]). The additional spots observed in the pattern (indicated with white arrows) can be attributed to the presence of twins in InAs QDs. It is important to note that at low temperature, the spots related to InAs QDs appear matched with the streaks of GaAs surface. We can conclude that at this stage of the growth, InAs QDs are lattice matched with GaAs buffer layer and consequently that InAs QDs are under strain, despite the high mismatch (7.2 %) between the lattice parameter of GaAs and InAs.

### Annealing

After the annealing step at 300 °C, (2×2) reconstruction on GaAs(111)A surface is again clear, due to removal of As excess accumulated during the As irradiation, while the spots related to InAs QDs are slightly shifted towards specular beam (see Fig. 1d). Calculating the ratio between spacing of GaAs streaks and the one of InAs spots, we can estimate a different lattice parameter of about 7 %, corresponding to almost fully relaxed InAs. This change can be related to the nucleation of dislocation at the interface between InAs and GaAs driven by the thermal energy added to the system by annealing procedure.

In scientific literature, it is reported that InAs layers on GaAs(111)A grow in planar mode [19, 20] instead of Stranski-Krastanow mode as reported for the case of InAs on GaAs(001). This behavior is due to strain relaxation induced by the introduction of dislocations at the interface between InAs and GaAs. The presence of these defects is expected to affect quality and optoelectronic properties of InAs QDs. The formation of strained (and consequently non–dislocated) InAs islands on GaAs(111)A was observed for low InAs coverage in [18, 20] and in our experiments is observed until substrate temperature is maintained low.

**Fig. 3:**
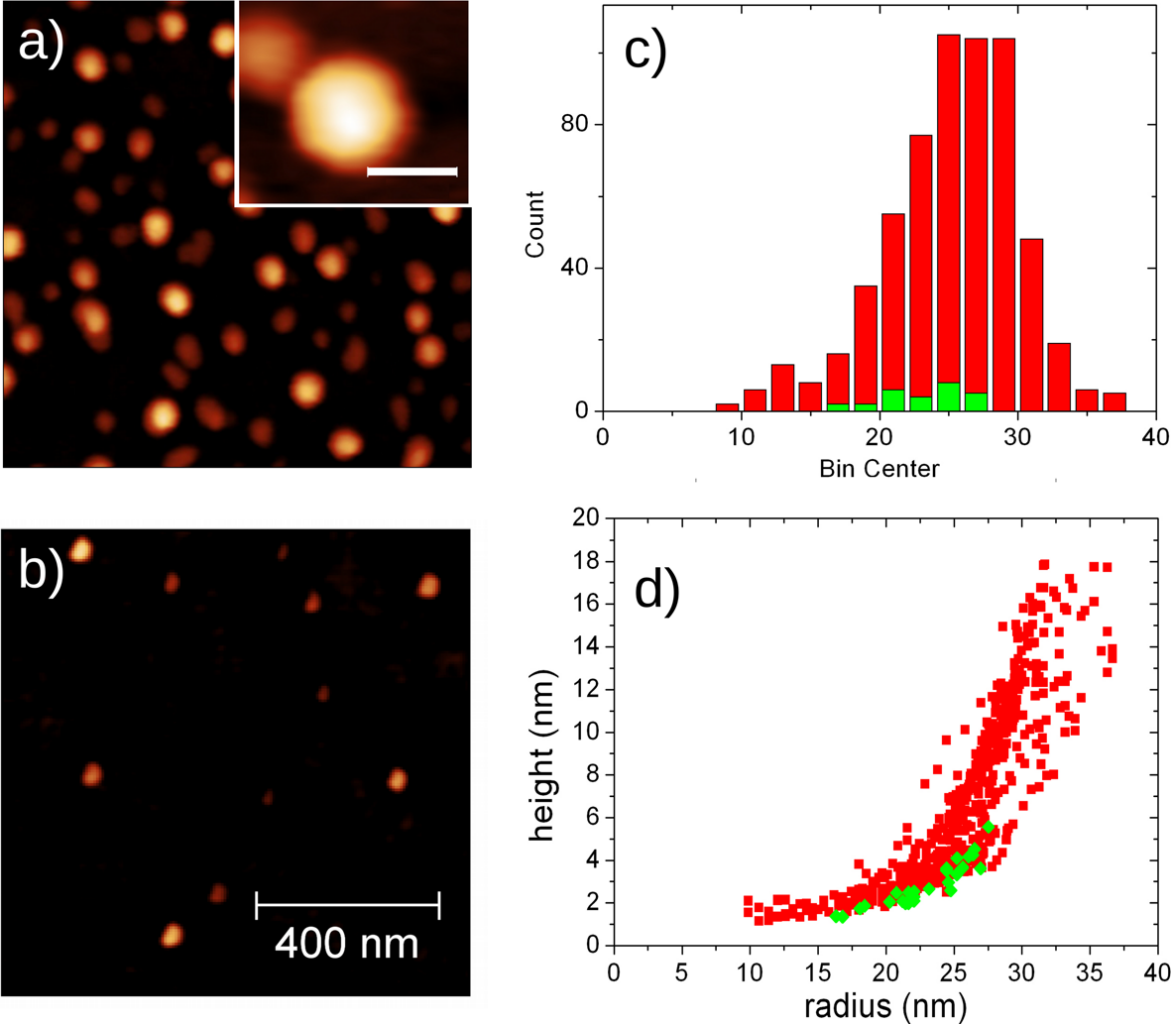
1×1*μ* *m* ^2^ AFM scan on surface of samples L1 (**a**) and H2 (**b**). In inset, magnification of a single InAs QD (*white bar* of inset corresponds to 40 nm). In **c**, radius distribution of InAs QDs is reported (*red bars* for sample L1 and *green bars* for sample H2). **d** shows size distribution of InAs QDs on the two samples. Each dot is reported as a *red square* (sample L1) and *green diamond* (sample H2)

The thermal annealing process is also affecting the shape and the size of InAs QDs. Figure 3a shows an AFM image of a 1×1*μm*^2^ area on samples L1. Inset of panel Fig. 3a shows magnification of a single InAs QD. The shape is hexagonal as reported for GaAs QDs arsenized at low temperature on (111)A surface [22]. Total volume of InAs QDs calculated from AFM scans is in good agreement with the volume calculated from the amount of deposited In, as reported on Table 1. This means that on sample L1, almost all deposited In is incorporated inside InAs QDs. Figure 3b shows an AFM image of a 1×1*μm*^2^ area on samples H2, grown with the same recipe and depositing the same amount of In to form InAs QDs, but with different temperature for the annealing (300 °C for sample L1 and 450 °C for sample H2). As reported in Table 1, the QD density on sample H2 is 50 times lower than the density on sample L1 and only ∼3 % of deposited In is incorporated in InAs QDs. Figure 3c, d shows a comparison between size distribution of InAs QDs on samples L1 and H2. The profiles observed for radius distributions (Fig. 3c) are, to a good approximation, Gaussian. The mean value of radius is quite similar for both samples (25.1 nm for sample L1 and 22.9 for sample H2), but standard deviation and aspect ratio (see Table 1) decrease by annealing InAs QDs at higher temperature. Aspect ratio decreases from 0.123 of sample L1 to 0.062 of sample H2. Standard deviation is reduced from 4.9 nm of sample L1 annealed at lower temperature, to 3.1 of sample H2 annealed at higher temperature. These differences between the two samples can be ascribed to increased diffusion length of In when annealing temperature is increased from 300 to 450 °C. This effect was already described for Ga and In on (100) surface in [39, 40] and [41], respectively. It can be explained considering that, by increasing annealing temperature, an increasing number of thermally activated In ad–atoms is generated due to bond breaking. Such ad–atoms diffuse following the equation [40]

(1) $$ \ell = \sqrt {D_{0} \exp\left(-E_{A}/k_{B}T\right) \frac{N_{s}}{J_{\text{As}}}}   $$

where *D*_0_ is the diffusivity prefactor, *E*_*A*_ the activation energy for diffusion, T the substrate temperature, *N*_*s*_ the number of surface sites, and *J*_*As*_ the arsenic flux.

Considering the volume of InAs QDs to be constant, as re–evaporation of In up to 450 °C is negligible, when diffusion length of In is increased, the radius of each dot is increased and the height is decreased. If we consider pyramidal QDs with an hexagonal base, from the definition of volume of the pyramid and of aspect ratio, we expect a relation due to annealing process

(2) $$ \frac{AR'}{AR}=\frac{r^{3}}{(r+\ell)^{3}}   $$

where AR and AR’ are aspect ratios before and after the annealing, *r* is the initial radius of the QD (equal to base edge of the hexagonal pyramid) and *ℓ* the diffusion length of In ad–atoms as defined in Eq. 1. The effect of the annealing process is then to reduce the height and increase the radius of the QDs and is more evident on smaller dots. With *r*_dot_≲*ℓ*, we expect a reduction of aspect ratio of eight times or more. In these conditions, it is then quite easy to understand that smaller dots can be flattened to a single monolayer height on the surface with the formation of a InAs 2–D layer on the surface, as observed in [42]. The decrease observed in mean aspect ratio for InAs QDs on samples L1 and H2 confirms this model, as reported in Table 1. Also an increase of mean radius is expected, but we have to consider that the formation of a layer originated by flattening of InAs QDs explains the reason why the centre of radius distribution is not apparently increased from sample L1 to sample H2, as reported in Fig. 3c. We have to consider that on sample H2, InAs QDs are partially buried by the InAs layer formed on the surface. The flattening of the smaller dots is also confirmed by the reduction in density of InAs QDs observed in Table 1 for higher annealing temperature (wider *ℓ*) and decreasing amount of In deposited (smaller mean radius of the dots). As shown by the data presented in Fig. 3d, the increased diffusion length leads to the formation of InAs QDs with lower aspect ratio [39] and to completely flatten smaller dots present on surface.

**Fig. 4:**
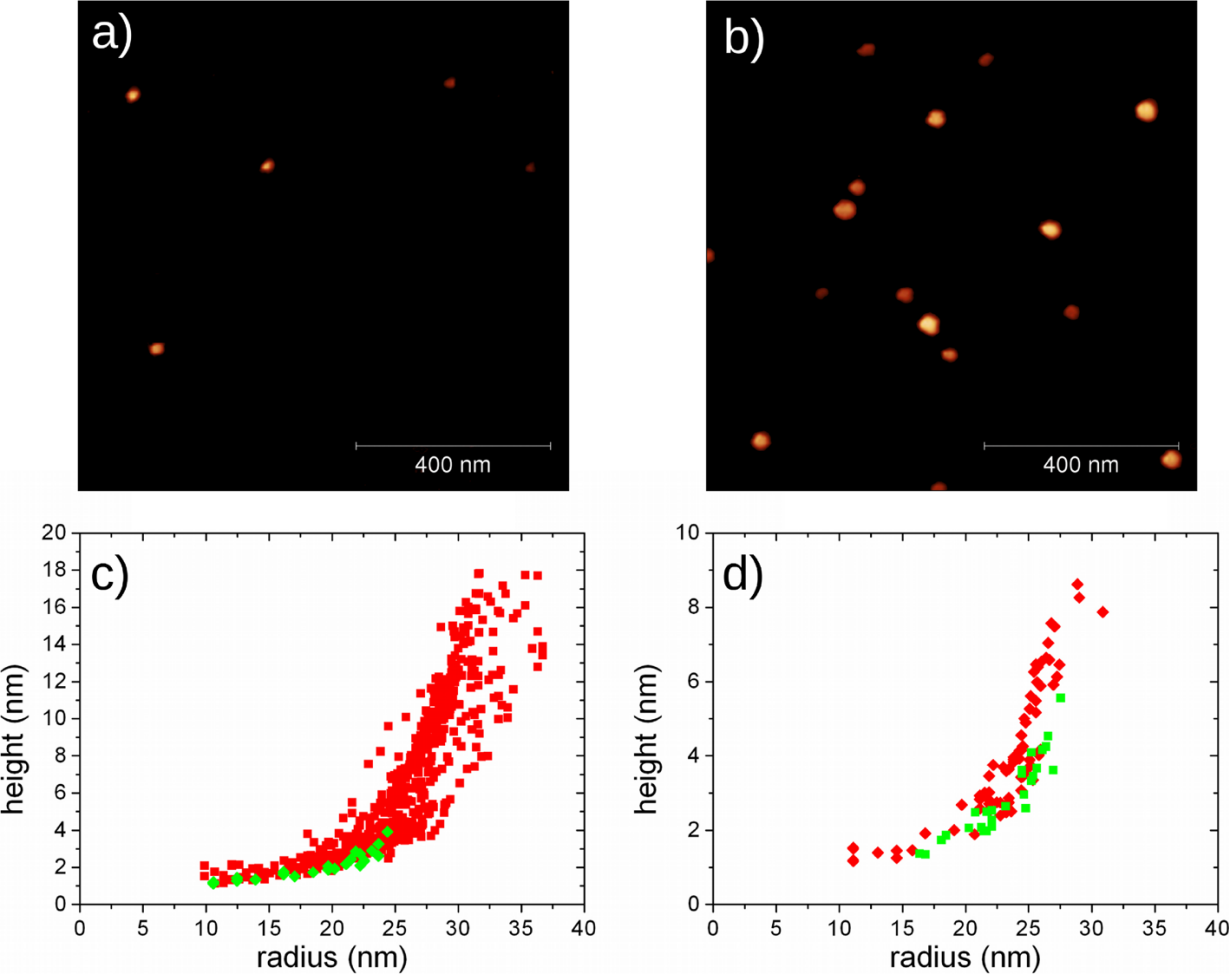
1×1*μ* *m* ^2^ AFM scan on surface of samples L2 (**a**) and H1 (**b**). Size distribution of InAs QDs grown in series L (**c**) and H (**d**). Each *dot* is reported as a *red square* (sample L1 on **c** and sample H1 on **d**) and *green diamond* (sample L2 on **c** and sample H2 on **d**)

This behavior is confirmed by the two series L and H, annealed at different temperatures (300 °C for L series and 450 °C for H series). In each series, three different amounts of In were supplied to the surface. In fact, decreasing the amount of deposited In for nano–crystal formation, we leave the density of QDs unchanged, but we decrease the initial size of each InAs QDs. Figure 4a, b shows AFM images of 1×1*μm*^2^ area on samples L2 and H1, respectively. In Fig. 4c, d, the size (radius–height) of each dot found in a 2×2*μm*^2^ AFM scan is reported. In Fig. 4c, d, each red square and green diamond stands for a InAs QD on sample L1 (H1) and L2 (H2), respectively. Reducing the initial amount of In, the dots after annealing become smaller and lower, and aspect ratio and density of QDs are decreased (see also Table 1). Below, a certain critical value, InAs QDs are completely flattened, as observed on samples L3 and H3. As reported in Table 1, this critical value is changing with annealing conditions, being in 0.4–0.6 ML range for L series (low annealing temperature) and in 1.5–1.0 MLs range for H series (high annealing temperature). As expected from diffusion length (Eq. 1), the critical amount of In increases upon increase of annealing temperature.

It is also interesting to note that size distribution of QDs is reduced by thermal annealing. As reported in Table 1, the radius of QDs on samples annealed at higher temperatures has a standard deviation on radius reduced by a factor ∼1.6.

## Conclusions

We reported the study on formation of InAs QDs grown by droplet epitaxy on GaAs (111)A surface, performed by a mean of RHEED pattern and of AFM analysis. We demonstrated that InAs QDs arsenized at low temperature are lattice matched with GaAs substrate and become almost fully relaxed when substrate temperature is increased with the insertion of dislocations. We also studied the effect of annealing on density and aspect ratio of InAs QDs, showing that increasing annealing temperature, size dispersion is reduced, while density and aspect ratio decrease up to complete flattening of smaller dots.
